# Prevalence of Dry Eye Disease Among Medical Students and Its Association with Sleep Habits, Use of Electronic Devices and Caffeine Consumption: A Cross-Sectional Questionnaire

**DOI:** 10.2147/OPTH.S397022

**Published:** 2023-04-03

**Authors:** Luai Abu-Ismail, Mohammad T Abuawwad, Mohammad J Taha, Almu’atasim Khamees, Dima Y Abu Ismail, Mohammad Sanwar, Yaqeen Al-Bustanji, Abdulqadir Nashwan, Omran Hamdan Alameri, Hamzeh Mohammad Alrawashdeh, Hashem Abu Serhan, Jocob Abu-Ismail

**Affiliations:** 1 Department of Ophthalmology, Islamic Hospital, Amman, Jordan; 2 Department of Clinical Medicine, Cairo University, Cairo, Egypt; 3 Department of General Surgery, King Hussein Cancer Center, Amman, Jordan; 4 Department of Clinical Medical Sciences, Faculty of Medicine, Hashemite University, Zarqa, Jordan; 5 Department of Medicine, Jordan University Hospital, Amman, Jordan; 6 Department of Clinical Medicine, School of Medicine, The University of Jordan, Amman, Jordan; 7 Department of Nursing Education and Research, Hamad Medical Corporation, Doha, Qatar; 8 Department of Pathology and Public Health, Faculty of Veterinary Medicine, Jordan University of Science and Technology, Irbid, Jordan; 9 Sharif Eye Centers, Irbid, Jordan; 10 Department of Ophthalmology, Hamad Medical Corporation, Doha, Qatar; 11 Department of Ophthalmology, Specialty Hospital, Amman, Jordan

**Keywords:** caffeine, dry eye, medical students, screen exposure, sleep quality

## Abstract

**Introduction:**

Dry eye disease (DED) is a common and multifactorial disease of the ocular surface which causes visual disturbance and feelings of discomfort among patients. The prevalence rate among medical students is an important issue to consider. This study investigates the relationship between caffeine consumption, sleeping habits, use of electronic devices, and DED among a convenient sample of medical students in Jordan.

**Methods:**

This cross-sectional online survey enrolled medical students from all six medical schools in Jordan. The questionnaire, which was shared via social media platforms, assessed socio-demographics, caffeine consumption amounts and patterns, sleep quality, and the use of electronic devices and their relation to ocular discomfort, DED, and related symptoms. The ocular surface disease index (OSDI) questionnaire was also administered to quantify the symptoms of DED.

**Results:**

A total of 1223 students participated in this study (RR=24.46%); 64% were females, and 43% were in their clinical placement years. Of the participants, 317 (25.92%) had normal eyes, and 906 (74.08%) had symptomatic DED. Of the students, 1206 (98.6%) used electronic devices directly before bed, and only 399 (32.62%) used blue-light-protective glasses. Lower DED risk was linked to male gender (OR=0.535, 95% CI 0.392–0.73, p <0.01), clinical years of medical school (OR=0.564, 95% CI 0.424–0.75, p<0.01). Poor sleep quality corresponded to more incidence of DED, regardless of sleeping for 5–6 hours (OR=3.046, 95% CI 1.299–7.139, p=0.01) or for less than 5 hours (OR=3.942, 95% CI 1.824–8.519, p<0.01). Also, caffeine consumption only marginally affected its incidence, but the results were statistically insignificant.

**Conclusion:**

Female gender, basic science years, and spending more than 6 hours looking at screens were significantly associated with symptomatic DED. Caffeine consumption did not pose any significant risk to the incidence of DED.

## Introduction

Dry eye disease (DED) is a complex multifactorial disorder, characterized by homeostatic disturbances of the ocular surface and tear film.^1^ It is among the most commonly encountered chronic ophthalmic conditions in clinical practice, with adult population prevalence estimates ranging from 5% to 50% in different parts of the world.^1^,^2^ DED impacts patients’ quality of life as it causes them pain, lowers their vitality and hinders their capacity at performing several tasks like driving and reading, conclusively resulting in a reduction in their quality of life and their competence in their place of work.^3^

Medical students face a lot of stress during their educational journey. They try to accommodate these stressful life conditions by developing habits that may be unhealthy, which affect their performance. In addition to the prolonged use of laptops, tablets, smartphones, and projectors for academic purposes, university students, whether medical or non-medical, are more likely to consume caffeinated beverages.^4^,^5^ The average daily caffeine intake varies from country to country, but it has been estimated that a 70-kg-adult would consume around 200 to 300 mg of caffeine per day in most Western countries.^6^ Furthermore, medical students may not consider sleep as a top priority in the context of their academic requirements as they reduce their sleeping time to have extra hours for studying and workload. It is reported that 51% and 59% of the medical students had poor sleep quality in the United States^7^ and Lithuania,^8^ respectively. In light of this, we aimed to report the prevalence of DED among medical students in Jordan and discuss its association with sleep habits and caffeinated beverages consumption.

## Methodology

### Sample and Data Collection

This is a descriptive cross-sectional study. The study population was made up of medical students (all years) from all Jordan’s six medical schools. The students who participated in the study received an online questionnaire between March 28 and April 6, 2020. According to the Checklist for Reporting Results of Internet E-Surveys (CHERRIES),^9^ the questionnaire was provided in English as it is the official language of teaching in all medical schools in Jordan. Google Forms platform was used to build the online questionnaire and shared through the university official e-mail group. The survey link was circulated for more than 5000 students in all the six medical schools.

Twenty-nine closed-ended questions were included in the survey, which was divided into four main sections: 1) sociodemographic characteristics; 2) caffeine consumption amount and pattern; 3) sleep quality and pattern, usage of electronic devices and its relationship to ocular discomfort; and 4) DED and associated symptoms. Additionally, the Ocular Surface Disease Index (OSDI) questionnaire, which is intended to quickly assess symptoms of dry eye illness and ocular irritation over the previous week.^10^ The OSDI elicited a good specificity (0.83) with a sensitivity (0.60).^11^

A consent statement about voluntary involvement in the study was included at the start of the questionnaire. The Institutional Review Board of the Jordanian Ministry of Health gave its approval number (1539) in accordance with the principles of the Helsinki Declaration. The sample size was calculated using Epi Info^TM^ v.7.2.4.0 (Epi Info^TM^, version 7.2.4.0, a database and statistics program for public health professionals. CDC, Atlanta, GA, USA, 2011) considering a previous study performed in Jordan with a sample population proportion of 68%.^12^,^13^ Using a cross-sectional study design, where n = required sample size (n = *Z* (α/2) 2 pq/d2), we calculated the sample size based on the following parameters:

Prevalence of 68%, precision of 0.04, 99% confidence interval (CI), and 5% margin of error. We estimated 518 as the minimum sample size required to represent the true population. A convenience sampling size (n =1223) was used in this study.

In our survey, each student was asked to check their most frequent drinks from a list of common drinks in Jordan, then these drinks were matched to their caffeine content.^14^,^15^ Multiple choices were allowed. Then, each student provided the frequency of drinking a caffeinated beverage, and the cup size they most frequently use (8, 12 or 16 ounces). To obtain the average caffeine intake per student, the average caffeine content for drinks was calculated per student, then multiplied by the frequency of drinking and by cup size. The result was an approximation for daily caffeine intake for each student.

$${\rm{Daily\ Average\ Caffeine\ Intake}}={\rm{{{Average\ Caffeine\ Content\ of\ Drinks} \over {Number\ of\ chosen\ drinks}}}} \times {\rm{Frequency\ of\ drinking\ Caffiene}} \times {\rm{Preferd\ Cup\ Size}}$$

Equation 1: Calculation of mean daily caffeine intake per student per day

### Statistical Analysis

Frequencies and proportions were used to summarize the data. Comparisons between categorical variables were analyzed using the Pearson Chi-square test. In order to examine the factors linked to dry eye disease, binary logistic regression was performed. At a p-value of 0.05, statistical significance was deemed to exist. The Social Sciences Statistical Program was used to conduct the statistical analysis (IBM SPSS Corp, SPSS Statistics ver. 26, USA).

## Results

A total of 1223 undergraduate medical students completed the online questionnaire (RR=24.46%). All students were 18–24 years old, 783 (64%) were females and 440 (36%) were males. The participants were medical students from the different years of medical school distributed between basic levels 693 (57%) and clinical level 530 (43%). Most students were non-smokers 1038 (85%). The attitude regarding the consumption of caffeine and sleep was clear as most of them increased caffeine consumption during examination period with 76% (927) and 74% of the students went to bed after 12:00 AM (Table 1).

**Table 1:** Description of Socio-Demographic Distribution of Students

Variable	Number (%)
**Gender**	
Female	783 (64)
Male	440 (36)
**Year of study**	
First	310 (25)
Second	203 (17)
Third	180 (15)
Fourth	203 (17)
Fifth	202 (17)
Sixth	125 (10)
**Study level**	
Basic	693 (57)
Clinical	530 (43)
**Smoker**	
No	1038 (85)
Yes	185 (15)
**Do you increase caffeine consumption during exams?**	
No	296 (24)
Yes	927 (76)
**Do you use your phone or any electronic device before going directly to bed?**	
No	17 (1.4)
Yes	1206 (98.6)
**At what time do you usually go to bed at night?**	
After 12.00 AM	900 (74)
Before 12.00 AM	323 (26)
**How often do you use the eye lubricant?**	
Never	860 (70)
When I feel that I need to use it	274 (22)
Regularly	30 (2)
Twice a day	35 (3)
3 times a day	24 (2)

The level of study significantly affected the sleeping quality as 422 (34.51%) of basic level students evaluated their sleep quality poor to neutral. It was noted that 259 (21.2%) of the medical students suffered from poor quality of sleep with 13.41% in the basic level compared to 7.77% in clinical level (P<0.01). Interestingly, regular eye checkups were not common among them as 37.53% (459) stated that they do that (P<0.01) with a majority in the basic level 283 (23.14%) compared to those in clinical level 176 (14.39%) (Table 2).

**Table 2:** Study Level and Other Factors Affecting the Eye Health

		Study Level			P-value
		Basic	Clinical	Total	
**Sleep Quality**	Poor	164 (13.41%)	95 (7.77%)	259 (21.2%)	<0.01
	Neutral	258 (21.1%)	173 (14.15%)	431 (35.2%)	
	Good	271 (22.16%)	262 (21.42%)	533 (43.6%)	
**Do you use contact lenses?**	No	625 (51.1%)	457 (37.37%)	1082 (88.47%)	0.03
	Yes	68 (5.56%)	73 (5.97%)	141 (11.53%)	
**Do you use blue light protective glasses when you use electronic devices?**	No	476 (38.92%)	348 (28.45%)	824 (67.37%)	0.26
	Yes	217 (17.74%)	182 (14.88%)	399 (32.62%)	
**Do you visit an ophthalmologist regularly to check your eye’s health?**	No	410 (33.52%)	354 (28.95%)	764 (62.47%)	<0.01
	Yes	283 (23.14%)	176 (14.39%)	459 (37.53%)	
**Do you use eye lubricant drops?**	No	494 (40.39%)	364 (29.76%)	858 (70.15%)	0.32
	Yes	199 (16.27%)	166 (13.57%)	365 (29.84%)	
**Total**		693 (56.7%)	530 (43.3%)	1223 (100%)	

Female students reported higher OSDI numbers, as 487 (39.82%) of them had moderate-to-severe OSDI results (p<0.01). Basic level students showed a higher prevalence of severe OSDI number 320 (26.17%) (p<0.01). One interesting finding was that 893 (73.01%) students who use their phone directly before bed reported OSDI numbers corresponding to mild-to-severe dry eye (p=0.98). High exposure to screens was common among students with severe OSDI reading, as 363 (29.68%) students exposed to screens over 6 hours daily developed severe DED (p<0.01) (Table 3).

**Table 3:** Pearson Chi-Square Test of Association Between OSDI Results and Socio-Demographic Factors

		OSDI Number (%)					P-value
		Normal	Mild	Moderate	Severe	Total	
**Gender**	Female	162 (13.25%)	134 (10.96%)	104 (8.5%)	383 (31.32%)	783 (64.03%)	<0.01
	Male	15 (12.67%)	103 (8.42%)	47 (3.84%)	135 (11.04%)	440 (35.97%)	
**Study level**	Basic	149 (12.18%)	135 (11.04%)	89 (7.28%)	320 (26.17%)	693 (56.67%)	<0.01
	Clinical	168 (13.74%)	102 (8.34%)	62 (5.07%)	198 (16.19%)	530 (43.34%)	
**Do you use your phone or any electronic device before going directly to bed?**	No	4 (0.33%)	3 (0.25%)	2 (0.16%)	8 (0.65%)	17 (1.4%)	0.98
	Yes	313 (25.59%)	234 (19.13%)	149 (12.18%)	510 (41.7%)	1206 (98.6%)	
**Average time of exposure to screens daily**	<4 hours a day	32 (2.62%)	23 (1.88%)	12 (0.98%)	38 (3.11%)	105 (8.59%)	<0.01
	4–6 hours a day	98 (8.01%)	85 (6.95%)	43 (3.52%)	117 (9.57%)	343 (28.05%)	
	>6 hours a day	187 (15.29%)	129 (10.55%)	96 (7.85%)	363 (29.68%)	775 (63.37%)	
**Do you use blue light protective glasses when you use electronic devices?**	No	250 (20.44%)	181 (14.8%)	98 (8.01%)	295 (24.12%)	824 (67.37%)	<0.01
	Yes	67 (5.48%)	56 (4.58%)	53 (4.33%)	223 (18.23%)	399 (32.62%)	
**Total**		317 (25.92%)	237 (19.38%)	151 (12.34%)	518 (42.36%)	1223 (100%)	

Students varied in their caffeine consumption. A total of 563 (46%) students consumed low amounts of caffeine, of whom 156 reported normal OSDI numbers, while 24.2% of the students with severe dry eye ingested moderate-to-high amounts of caffeine daily (p=0.095). Majority of students (435, 35.6%) had their last caffeinated drink more than 4 hours before bedtime. Students differed in their sleep time, with only 176 (14.4%) sleeping for less than 5 hours per night and 129 (10.5%) had high OSDI-number ranging from mild to severe (p=0.114). The quality of sleep-in students was also assessed, and of 317 (25.9%) students who reported normal eyes, most (179, 14.6%) reported good quality sleep (p<0.01) (Table 4).

**Table 4:** Pearson Chi-Square Test of Association Between OSDI Results and Caffeine Consumption and Sleep Quality Factors

		OSDI Number (%)					P-value
		Normal	Mild	Moderate	Severe	Total	
**Mean daily caffeine intake**	Low	156 (12.8%)	112 (9.2%)	73 (6%)	222 (18.2%)	563 (46%)	0.095
	Moderate	115 (9.4%)	89 (7.3%)	53 (4.3%)	181 (14.8%)	438 (35.8%)	
	High	46 (3.8%)	36 (2.9%)	25 (2%)	115 (9.4%)	222 (18.2%)	
**At which time do you have your last caffeinated drink before going to sleep?**	Directly before going to bed	10 (0.8%)	8 (0.7%)	6 (0.5%)	43 (3.5%)	67 (5.5%)	<0.01
	1–2 hours before going to bed	77 (6.3%)	71 (5.8%)	35 (2.9%)	165 (13.5%)	348 (28.5%)	
	3–4 hours before going to bed	92 (7.5%)	67 (5.5%)	50 (4.1%)	164 (13.4%)	373 (30.5%)	
	More than 4 hours before going to bed	138 (11.3%)	91 (7.4%)	60 (4.9%)	146 (11.9%)	435 (35.6%)	
**How many hours of actual sleep do you get at night?**	<5 hours	47 (3.8%)	25 (2%)	24 (2%)	80 (6.5%)	176 (14.4%)	0.114
	5–6 hours	85 (7%)	73 (6%)	42 (3.4%)	181 (14.8%)	381 (31.2%)	
	6–7 hours	126 (10.3%)	93 (7.6%)	51 (4.2%)	163 (13.3%)	433 (35.4%)	
	>7 hours	59 (4.8%)	46 (3.8%)	34 (2.8%)	94 (7.7%)	233 (19.1%)	
**Sleep quality**	Good	179 (14.6%)	110 (9%)	60 (4.9%)	184 (15%)	533 (43.6%)	<0.01
	Neutral	110 (9%)	84 (6.9%)	55 (4.5%)	182 (14.9%)	431 (35.2%)	
	Poor	28 (2.3%)	43 (3.5%)	36 (2.9%)	152 (12.4%)	259 (21.2%)	
**Total**		317 (25.9%)	237 (19.4%)	151 (12.3%)	518 (42.4%)	1223 (100%)	

Binary logistic regression was used to examine the effects of socio-demographic and medical factors on producing DED. Our sample had 906 (74.1%) students with DED and 317 (25.9%) with healthy normal eyes. Our data satisfied the binary logistic regression assumptions, as it has independent observations, without perfect multicollinearity, and had no continuous predictor. The model was shown to be statistically significant compared to the baseline at describing reasons of DED (p<0.001). It predicted DED in 75.8% of the students of whom 94.7% had DED and 21.8% were healthy. Our model predicts a significant impact of gender on of the occurrence of DED, as it is estimated that male students are less likely to have DED (OR=0.535, 95% CI 0.392–0.73, p<0.01). Another factor of significant impact was the study level of students, since clinical level students are at a favorable 0.564 odds of having DED compared to basic students (95% CI 0.424–0.75, p<0.01). It is noted that students who tend to use with blue light protection glasses had a higher incidence of DED (OR=1.798, 95% CI 1.281–2.525, p<0.01), and similarly, students who visit an ophthalmologist regularly were estimated to have higher DED risk, most likely due to an already symptomatic DED (OR=1.614, 95% CI 1.159–2.249, p<0.01). We multiplied the results of questions (Do you use eye lubricant?) with (How often do you use the lubricant?) to conclude a single factor that estimates the effect of using eye lubricants on the incidence of DED. Compared to non-users, students who use eye lubricant regularly reported more incidence of DED (OR=3.257, 95% CI 2.122–4.999, p<0.01). Sleep quality multiplied with hours of actual sleep at night had a similar effect as using eye lubricant. Sleeping less or having worse quality of sleep resulted in increasing the odds of having DED. However, poor sleep quality had more significant results in comparison to neutral sleep quality. In reference to good sleep for 7 hours or more (p<0.01), poor sleep for 5–6 hours a night increases the odds for having DED by 3.046 (95% CI 1.299–7.139, p=0.01) and a poor sleep for less than 5 hours increases it to 3.942 (95% CI 1.824–8.519, p<0.01). Increased consumption of caffeine corresponded to a higher incidence of DED. In comparison to students with low caffeine consumption, students who consumed caffeine in moderate-to-high rates reported higher risks of developing DED (OR=1.059, 95% CI 0.766–1.465, p=0.728) and (OR=1.016, 95% CI 0.649–1.591, p=0.944), respectively. However, these results are statistically insignificant. Using electronic devices directly before going to bed did not result in a significant effect on the prediction on DED (p=0.88), and in the same manner, time of exposure to screens was not of considerable effect in our model (Table 5).

**Table 5:** Binary Logistic Regression Estimating the Effects of Factors on the Occurrence of DED

Factor	Estimate	Odds Ratio	95% CI for EXP(B)		P-value
			Lower	Upper	
**Gender**					
Female*					
**Male**	−0.625	0.535	0.392	0.730	<0.01
**Study level**					
Basic*					
**Clinical**	−0.573	0.564	0.424	0.750	<0.01
**At which time do you have your last caffeinated drink before going to sleep?**					
Directly*					0.037
**1–2 hours**	−0.463	0.630	0.294	1.346	0.233
**3–4 hours**	−0.571	0.565	0.263	1.213	0.143
**>4 hours**	−0.885	0.413	0.193	0.884	0.023
**Do you use eyeglasses with protection from blue light when you use your electronic devices?**					
No*					
**Yes**	0.587	1.798	1.281	2.525	<0.01
**Do you visit an ophthalmologist regularly to check your eye’s health?**					
No*					
**Yes**	0.479	1.614	1.159	2.249	<0.01
**Do you use eye lubricant drops? × How often do you use the lubricant?**					
No* × Never*					<0.01
**Yes × When I feel the need to use it**	1.181	3.257	2.122	4.999	<0.01
**Yes × Regularly**	0.486	1.626	0.535	4.943	0.392
**Yes × Twice a day**	0.674	1.962	0.765	5.031	0.161
**Yes × 3 Times or more daily**	1.176	3.241	0.713	14.735	0.128
**How many hours of actual sleep do you get at night? × Sleep quality**					
>7 hours* × Good*					<0.01
**Less than 5 hours × Neutral**	0.371	1.449	0.954	2.201	0.082
**Less than 5 hours × Poor**	1.372	3.942	1.824	8.519	<0.01
**5–6 hours × Neutral**	0.328	1.388	0.879	2.193	0.160
**5–6 hours × Poor**	1.114	3.046	1.299	7.139	0.01
**6–7 hours × Neutral**	0.479	1.614	0.787	3.312	0.192
**6–7 hours × Poor**	0.818	2.267	0.893	5.753	0.085
**Mean daily caffeine intake**					
**Low**					0.938
**Moderate**	0.058	1.059	0.766	1.465	0.728
**High**	0.016	1.016	0.649	1.591	0.944
**Constant**	1.234	3.434			0.103

**Note**: *Reference category.

## Discussion

Medical students are a special group with special characteristics. Their lifestyle puts them under a great deal of stress, driving them to certain behaviors in order to cope, such as ingesting caffeinated drinks frequently and abusively using their electronic devices. In our study, we investigated the effects of these patterns of behavior on the eyes of medical students in Jordan, focusing on the incidence of DED in particular.

Our results showed that female gender and basic level of medical school are associated with a higher risk of developing DED. This result agrees with the epidemiology of DED as described in literature.^16^,^17^ These results were also reported by Al-Dolat et al^18^ in their study on DED in Jordanian students, where the authors reported that female or basic level students were at higher risk of DED by 2.4 and 2.2 odds, respectively. On the other hand, both our study and Al-Dolat et al study^18^ described a population and a sample size with a majority of females.

Our students varied in their caffeine consumption but most of them are considered low to moderate consumers. Also, most students ramped up their caffeine consumption during examination period with 76% (927), compared to another Jordanian study that reported that less than half of their participants (46%) consumed caffeine to wake more hours during examinations.^19^ Regarding the relationship between the amount of caffeine consumption and DED, 296 (24.2%) students with severe dry eyes ingested moderate-to-high amounts of caffeine daily (p = 0.095), which indicates that caffeine consumption may be one of the risk factors for dry eye; however, these results did not reach statistical significance.

Using phones directly before going to bed is also associated with higher OSDI scores, which indicates that it could be considered as a risk factor for DED. A study conducted by Wang and his team^20^ concurred with our results for female gender and increased digital screen exposure time, considering both as positive risk factors of DED. They also reported that increased caffeine consumption was a significant protective factor for DED; however, it is important to note that their population did not consist of medical students solely, and had a mean age of 41 ± 22.^20^ This difference between population can explain the varying results between our work and Wang study since medical students use caffeinated drinks to help them decrease their overall sleep, subjecting them to more incidence of DED, but the Wang study has a population whose consumption of caffeine does not affect their sleeping patterns. Our results showed that students differ in their sleep amount, with the majority of students (66.6%) sleeping between 5 and 7 hours, and (78.8%) having neutral to good sleep quality, and by using binary logistic regression, the results showed that sleeping less or having poorer quality of sleep result in an increased incidence of DED. These results were also found in many previous studies. A study from Japan found that sleep disturbances seem to be influencing factors of DED as 45% of the participants in the DED group reported poor sleep quality.^21^ Particularly, there were considerably greater risks for DED in mild and severe short-sleepers. Additionally, the Korean study noted that the addition of sociodemographic characteristics increased the likelihood of DED among mild and severe short-sleepers. Moreover, DED was twice as common in women as it was in men.^22^ Another study from the Netherlands was the first study to show that the relationship between poor sleep quality and dry eye is present in all segments of the population, affecting adults of all ages and sexes. However, this study investigated this relationship from the opposite direction as it studied the effects of dry eye on sleep quality, meaning that the relationship between DED and sleep quality might be bidirectional.^23^ The decrease in sleep time could cause DED by many different mechanisms. Physically, people who sleep for shorter amounts of time keep their eyes open longer. As a result, ocular exposure to dry environmental conditions is higher than it is for people who get enough sleep. Chemically, osmolality on the surface of the eye is hypothesized to decrease during sleep. Tear hyperosmolarity harms ocular surface epithelial cells, causing inflammation.^24^ As a result of inflammation, apoptosis of epithelial and goblet cells takes place and results in a decrease in production of mucins that are essential for the integrity of the tear film.^25^

The strength of our study is that it has good statistical power because of its large sample size relative to the total number of Jordanian medical students. Also, in this survey, we included only a homogeneous group of Jordanian medical students, which has the benefit of comparing similar age groups, habits, study hardness, and other environmental factors such as climate. Furthermore, this study discusses both quantity and quality of sleep with investigation into many factors and habits that affect sleep, in a trial to detect the worst practices and increase awareness about them. On the other hand, the limitations of this study were as follows: firstly, although this cross-sectional study describes the relationship between dry eye and sleep quality, caffeine consumption, and many other factors, it did not provide a full analysis of the correlation between these factors, since such process would overcomplicate the study; however, it addressed the general understanding of the issue and the students’ habits which should be taken in consideration as a possible factor of having DED. Secondly, the responses of these studies are subjective responses to sleep parameters and DED symptoms without a specific medical examination, which could differ from person to person. Thirdly, clinical evaluation of the type of DED would give more insight toward developing better understanding of the correlation between the factors addressed earlier which was a limitation in our study. Future studies investigating the mechanisms by which caffeine might cause DED are needed. Also, further elaboration of the relationship between them requires more research.

## Conclusion

The current study discovered that, among Jordanian medical students, shorter sleep durations, low sleep quality, basic level of studying, female gender, and using a phone right before bed, are strongly connected with DED. On the other hand, good quality and quantity of sleep, using eye lubricants, and wearing glasses were linked to lower OSDI scores, suggesting that they may act as a preventative measure against DED. Finally, the findings of the current study may shed light on potential treatment strategies and raise awareness among medical students in particular and the general public regarding the behaviors and habits that lead to DED.

## Data Availability

All data generated or analyzed during this study are included in this article. Further enquiries can be directed to the corresponding author.
